# *Notes from the Field*: Influenza A(H3N2) Outbreak Following a School Event — Los Angeles, California, March 2022

**DOI:** 10.15585/mmwr.mm7122a4

**Published:** 2022-06-03

**Authors:** Lello Tesema, Dominique Sullivan, Marifi Pulido, Elizabeth Traub, Jose Escobar, Leo Moore, Nicole Green, Peera Hemarajata, Maria Cruely, Rachel Civen, Alicia El-Togby, Garin Ohannessian, Sylvia Silas, Rosita San Diego, Dawn Terashita, Sharon Balter, Prabhu Gounder

**Affiliations:** ^1^Los Angeles County Department of Public Health, Los Angeles, California; ^2^California Public Health Laboratory, Los Angeles County Department of Public Health, Los Angeles, California.

On March 22, 2022, an outbreak of acute respiratory illness among attendees of an off-campus school banquet was reported to the Los Angeles County Department of Public Health (LACDPH). A total of 177 students and seven teachers had attended the banquet 3 days earlier. By March 21, illness with signs and symptoms that included fever, cough, headache, and fatigue was reported by 72 (41%) students. Four students sought treatment at an urgent care facility; none were hospitalized. The median interval from the banquet to symptom onset was 47 hours (range = 14–91 hours). Because of the high attack rate, school administrators closed the school to in-person attendance on March 21. LACDPH obtained a line list of all banquet attendees, developed a survey to ascertain symptoms and exposures, offered testing for respiratory pathogens including SARS-CoV-2 using a multiplex polymerase chain reaction assay (BioFire Diagnostics, LLC), and conducted an environmental assessment of the event hall.

Among the 184 attendees, 128 (63%) completed the survey, and 174 (95%) completed testing for respiratory pathogens (Table). Among those tested, 56 (32%) received a positive test result for influenza A(H3N2). The median interval from symptom onset to testing was 4 days (range = 0–11 days). SARS-CoV-2 was not detected among any of the tested participants. Of the 25 persons who responded regarding influenza vaccination status, four (16%) reported having received influenza vaccine before the school event, and 21 (84%) reported that they had not been vaccinated. Universal mandates regarding COVID-19 mitigation measures (i.e., mask use and physical distancing) had been lifted before the date the banquet occurred. Environmental assessment of the event space did not reveal any pertinent violations (e.g., issues with ventilation or overcrowding).

**TABLE:** Characteristics of attendees of a school banquet associated with an influenza A(H3N2) outbreak (N = 174)* — Los Angeles County, California, March 2022

Characteristic	Influenza test result,^†^ no. (%)		
	Total (N = 174)	Positive (n = 56)	Negative (n = 118)
**Age, yrs, median (range)**	16 (11–66)	15 (14–18)	17 (11–66)
**Sex**			
Male	89 (51)	39 (70)	50 (42)
Female	85 (49)	17 (30)	68 (58)
**Time from exposure to symptom onset, hrs, median (range)**	—	47 (14–91)	—
**Results of respiratory pathogen testing**			
SARS-CoV-2	13 (7)	0 (—)	0 (—)
Other pathogens	13 (7)	4^§^ (7)	13^¶^ (11)
Symptoms or fever**			
**Total**	**32 (18)**	**16 (29)**	**16 (14)**
Fever**	27 (16)	11 (69)	16 (100)
Cough**	30 (94)	14 (88)	16 (100)
Sore throat**	30 (94)	14 (88)	16 (100)
Fatigue**	26 (81)	14 (88)	13 (81)
Chills**	26 (81)	13 (81)	13 (81)
Headache**	25 (78)	13 (81)	12 (75)
Body aches**	24 (75)	13 (81)	11 (69)
**Influenza vaccination status**			
**Total^††^**	**25 (14)**	**15 (27)**	**10 (8)**
Vaccinated	4 (16)	1 (7)	3 (30)
Not vaccinated	21 (84)	14 (93)	7 (70)

* Of the 184 attendees, 174 were tested for respiratory pathogens.

^†^ Using multiplex polymerase chain reaction assay (BioFire Diagnostics, LLC).

^§^ Human rhinovirus/enterovirus (one), parainfluenza virus 2 (one), OC43 coronavirus (one), and 229E coronavirus (one).

^¶^ Human rhinovirus/enterovirus (six), parainfluenza virus 2 (four), 229E coronavirus (two), and 1 HKU1 coronavirus (one). Some persons received positive test results for more than one virus.

** Among respondents reporting fever or symptoms consistent with influenza. The total number of survey respondents who reported symptoms (32) is fewer than the total who reported symptoms to school administration (72).

**^††^** Among respondents who received multiplex polymerase chain reaction testing and provided information about influenza vaccination status.

LACDPH concluded that the outbreak was caused by influenza A(H3N2) virus. Although influenza activity has been lower this season than during seasons preceding the COVID-19 pandemic, large influenza outbreaks have been reported during the past year (*1*). Three co-occurring factors likely contributed to this large outbreak. First, influenza activity in the community was increasing at the time of this outbreak (the percentage of respiratory specimens testing positive for influenza at local sentinel laboratories had approximately tripled, from 0.9% during the week ending February 12, 2022, to 3.2% during the week ending March 19, 2022). Second, this increase in influenza activity coincided with the cessation of LACDPH mandates for face masks and physical distancing (March 1, 2022); mask mandates were lifted at this school on March 14. Given that the influenza virus is transmitted primarily through aerosols, the absence of mask use likely accelerated the spread. Third, interim estimates of influenza vaccine effectiveness against illness caused by influenza A(H3N2) virus infection were low this season (*2*). LACDPH recommended that all students and staff members wear face masks for ≥1 week after onset of the last symptomatic case at the school and advised persons who receive a positive influenza test result to immediately seek influenza antiviral therapy.

These findings highlight the potential for influenza viruses to cause outbreaks of acute respiratory illness with high attack rates. Several states have reported recent surges in late-season influenza activity this year. Vaccination can prevent serious influenza-related complications and is recommended for all persons eligible to receive the vaccine. As COVID-19 preventive measures are lifted across the country, influenza virus infections should be considered as a potential cause of respiratory outbreaks. 

## References

[1] Delahoy MJ, Mortenson L, Bauman L, Influenza A(H3N2) outbreak on a university campus—Michigan, October–November 2021. MMWR Morb Mortal Wkly Rep 2021;70:1712–4. 10.15585/mmwr.mm7049e1 34882659 PMC8659183

[2] Chung JR, Kim SS, Kondor RJ, Interim estimates of 2021–22 seasonal influenza vaccine effectiveness—United States, February 2022. MMWR Morb Mortal Wkly Rep 2022;71:365–70. 10.15585/mmwr.mm7110a1 35271561 PMC8911998

